# Understanding the roles of economy and society in the relative risks of zoonosis emergence from livestock

**DOI:** 10.1098/rsos.231709

**Published:** 2024-07-17

**Authors:** Stephen Hinchliffe, Alex Blanchette, Kin Wing (Ray) Chan, Chris Degeling, Jody Emel, Melissa Leach, Ian Scoones, Michael Winter

**Affiliations:** ^1^ Geography University of Exeter, Exeter, Devon, UK; ^2^ Wellcome Centre for Cultures and Environments of Health, University of Exeter, Exeter, UK; ^3^ Anthropology, Tufts University, Medford, Massachusetts, USA; ^4^ Agricultural Science and Practice, Royal Agricultural University, Cirencester, UK; ^5^ School of Health and Society, University of Wollongong Faculty of the Arts Social Sciences and Humanities, Wollongong, New South Wales, Australia; ^6^ Geography, Clark University Graduate School of Geography, Worcester, Massachusetts, USA; ^7^ Anthropology, Cambridge Conservation Initiative, Cambridge, Cambridgeshire, UK; ^8^ Institute of Development Studies, Brighton, UK; ^9^ Centre for Rural Policy Research (CRPR), University of Exeter, Exeter, Devon, UK

**Keywords:** pandemics, risk, agriculture, social science

## Abstract

The emergence of zoonotic infections that can develop into pathogens of pandemic potential is a major concern for public health. The risks of emergence and transmission relate to multiple factors that range from land use to human–non-human animal contacts. Livestock agriculture plays a potentially significant role in those risks, shaping landscapes and providing hosts that can act as the source or amplifiers of emergent pathogens. The relative risks will be contingent upon the nature of those systems, with comparisons often made between intensive, indoor, biosecure systems and more extensive, outdoor, insecure systems. Microbiological, ecological and veterinary sciences provide useful entry points in specifying and modelling some of the relative risks. Yet, they often do so with little regard for social science inputs and by making assumptions about social and economic conditions. In this article, we respond to recent analyses of relative risks by raising the importance of social and economic drivers of risk. We chart social science insights and research that materially alter the zoonotic risks associated with livestock production. Our purpose is to emphasize the requirement for full appreciation of the social, economic and political components of zoonotic and pandemic risk.

## 1. Introduction

After the COVID-19 pandemic, interest in the origins of and risks associated with emerging infectious diseases (EIDs) was reignited, with spillover of zoonotic viruses receiving the most attention [1–5]. Potential pandemic pathogens (PPPs) can emerge and evolve within non-human animal populations and, very rarely, cross over to people. Most animals and people will have limited pathogen-specific immunity to de novo pathogens. Once viruses or other microbes adapt sufficiently to allow for repeated human infections and become transmissible between these new hosts, then the microbial conditions are met for a potential pandemic. Dense and well-connected urban settlements, impoverishment, poor access to healthcare, infodemics and limited pandemic preparedness make for a highly biocommunicable planet (a term we use to suggest not only the production and circulation of information on pandemics but also the interconnectivity and infectivity of human and non-human hosts) [6,7]. Given this all too real scenario, any attempt to dampen risks of emergence, prevent spillover, amplification and transmission of zoonotic viruses is to be welcomed. Reducing habitat disruption, altering agricultural and land-use practices and improving agricultural biosecurity (usually defined as preventing the incursion of pathogens into livestock [8]) all merit urgent attention. Given the centrality of food and agriculture, and in particular, livestock farming, to each of these components of risk, an assessment of the role of different forms of livestock production systems is the key to any future planning and policy. Bartlett *et al.*’s [9] recent analysis in the pages of this journal offers a useful perspective on contemporary and future risks. The authors present an assessment of zoonotic emergence risks to argue, contrary to some points of view [10–12], that intensifying livestock production offers the best available means to reduce those risks. This article adds important social science analysis to their research. We argue that without proper consideration of the social and economic drivers of risk, any analysis will remain partial and potentially misleading. We start by providing a brief overview of their approach before working through key social and economic considerations that need to be developed in future models and assessments.

## 2. The view from veterinary microbiology

Risk is a probabilistic technology designed to assess the likelihood of a known event (in this case, a potential pandemic pathogen becoming established in human populations). In an epidemiological setting, risk assessment often involves tracing the edges or links between network nodes (or vulnerable populations), and then estimating the likelihood of ‘microbial traffic’ [1] between those populations. Risks increase as wild habitats are disturbed, causing stress and dispersion of wild animals, and/or as human contacts increase (though it should be noted that cohabitation and coevolution of people, animal hosts and microbes have long histories, and it may be the forms of disturbance, rather than contact *per se*, that matters) [13]. Risks will be increased with an expansion in livestock numbers and biomass. Indeed, zoonoses have emerged within livestock systems in the past [14], while the increased probability of wild–domestic animal interactions enhances the chances of acquisition, amplification and transmission of EIDs [15]. Once these or other envisaged spillover events occur, and once animal–human and human–human transmission is established, the regular, frequent and long-distance transit of people, wild and domestic animals aids pathogen spread [16]. This basic schema of reservoirs, contact points, amplifiers and transmission routes allows modellers to build and assess future scenarios regarding pandemic prevention measures.

Taking the model as its point of reference, Bartlett *et al.* [9] sampled and prioritized existing literature and drew upon a multi-disciplinary expert review of zoonosis prevention [17], to present a systematic review of the relative EID risks of different livestock systems. The risks of future zoonosis emergence were estimated assuming a business-as-usual scenario concerning economic growth, rising incomes and animal-derived product consumption. The historical relationship between rising average incomes and increased animal livestock product consumption, particularly pronounced in rapidly growing economies, was assumed to hold for future years. It is not our intention to question this assumption at this juncture, though it is worth noting that dietary norms are historically constituted [18], culturally contingent [19], influenced by agrifood global business value chains [20] and matters for health as well as environmental intervention [21,22]. These debates apart, the aim was to determine how this assumed demand for livestock products could be met at least cost in terms of EID risks. The authors assessed two contrasting systems of production (each assumed to deliver the same total output). The key comparator was yield, or product per unit area, a common agricultural measure of intensity (one that notably excludes other qualities including nutritional values [23,24]). Using yield, and the associated land-take for production, a series of assumptions regarding EID risks could be inferred. For example, ‘Brazilian beef production is expected to rise by approximately 14% between 2000 and 2040. At current yields this would require approximately 140 000 km^2^ of additional cultivated pasture, while if production switched entirely to less intensive, lower yielding systems, this rises to 570 000 km^2^... [moreover] increasing pasture productivity by 70% of its carrying capacity, future demand could be met on 360 000 km^2^ less land than in 2000...’ [9, p. 2]. Given that, in this scenario, extensive pasture is assumed to involve more land-take, greater habitat disruption and increased likelihood of wild animal/livestock/human interactions, then greater EID risk can be posited. All other things being equal, intensive production would imply a lower risk of zoonosis. Of course, these risks may be offset by other factors (livestock density, genetic diversity and disease resistance), so the authors reviewed a series of variables for the two methods of delivering the projected tonnage of meat demand.

Having established the basic comparator (product yield per unit of area), the authors judged the effect of each production method on the likelihood of future spillover, take-off and transmission events. In summary, we can represent the authors’ basic distinctions and findings as follows (see table 1).

**Table 1.** Summary of veterinary and microbiological EID risks associated with intensification of livestock production. Green indicates probable reduction of risk, amber indicates uncertain, while red suggests probable increase in risks.

**characteristic**	**h**igh yield	**l**ow yield	effect on EID risk once higher yield systems predominate (negative (green), or less risk, −, or positive (amber and red), more risk +)	**c**omments (implied effects of intensification on EID risk)
biosecurity	high	low	−	increased biosecurity reduces inward and onward transmission of pathogens
livestock movements	low	high	−	high-yield farms operate as closed systems, breeding on-site and not trading stock
population size	low	high	−	for the same gross product, high-yield intensive systems require lower overall livestock numbers to meet demand
density	high	low	+ (?)	greater density of livestock can increase transmission, though this will depend on the extent to which the density of host populations affects selection for virulent strains and on-farm containment of batch populations
health and welfare	uneven	uneven	+ (?)	the assumption of low welfare associated with indoor systems and intense production may be empirically challenged, while low-yield outdoor systems also have significant health and welfare challenges
disease resistance	low	high	+	low growth rates and outdoor-reared animals might be expected to offer more resistance to infection challenges, though rates of vaccination as well as the uncertain effects of immune reactions on viral selection make this a less certain benefit
genetic diversity	low	high	+	low-yield systems will be expected to offer greater genetic diversity of livestock, though the effects on pathogen evolution are less certain
habitat quality	high	low	−	high-yield systems have lower land-take and so enable greater conservation possibility for wild and semi-wild landscapes
ecotone extent	low	high	−	ecotones (transition zones between natural and farmed landscapes) are assumed to be more extensive in low-yield systems, increasing ecological fragmentation and interspecies contact rates
microhabitat extent	low	high	−	microhabitat refers to niches within farm environments that attract and maintain wildlife; their extent is considered to be greater in low-yield more-extensive systems, increasing interspecies contact and encouraging generalist host species
livestock contact with wildlife	low	high	−	in indoor and intensive systems, livestock–wildlife interactions are expected to be minimized
human contact with wildlife hosts in ecotone	low	high	−	human contact with wildlife will be reduced as the ecotone is diminished
human contact with livestock	low	high	−	lower-yield systems are assumed to have higher labour requirements; automation reduces labour in high-yield systems, making contact less frequent

Following this logic, in almost all measures, intensity is associated with reduced risks of zoonoses. The exceptions are for livestock density, health and welfare, and disease resistance, though in each case the authors note variance and uncertainty. Increases in livestock and farm density (numbers of animals per unit area) may be offset by on-farm segregation that can disrupt transmission. Health and welfare may be enhanced in well-managed indoor production systems [25]. Greater genetic diversity of a global or regional herd is also often assumed to confer disease resistance and resilience, with network heterogeneity disrupting transmission. Monocultures, in contrast, would seem to be vulnerable to rapid and irretrievable collapse once a pathogen evolves for a homogeneic population. However, there are uncertainties here too, as livestock diversity may increase pathogen diversification, result in more asymptomatic infections, and enable further species jumps. The devil is clearly in the detail (how much diversity, what does this afford in terms of viral receptors and immune responses?) As the authors rightly note, it ‘is difficult to disentangle how these countervailing risk factors might play out, and hence the overall effect of livestock diversity on EID risk’ [9, p. 7].

In a period where the prevention of future pandemics, minimizing habitat disruption and safeguarding global food supplies should be key priorities for governments and international organizations, the resulting risk estimations would seem to provide a clear steer for state and private investment in improved and more intensive forms of livestock production. That said, there is a fundamental need for careful analysis to proceed in tandem with the incorporation of insights from economic and social research on food and farming before these conclusions can be verified or supported. We therefore structure our response using a range of social science research and highlight why the social sciences need to be involved in pandemic preparedness and response research and discussion. We start by suggesting disciplinary and other forms of expertise that are required for such analyses.

## 3. On multi-disciplinary work

Bartlett *et al.*’s analysis draws on a multi-disciplinary review of pandemic risks with expertise drawn from zoological, veterinary, microbiological, animal health, ecology and other cognate disciplines. Notable by its absence was expertise in economics, geography, anthropology, political ecology, ethics or other social science disciplines. This exclusion impacts upon the issues that were considered relevant to EID risks and the methods used in generating the review of existing knowledge. For the latter, systematic reviews, using subjective in-group criteria for search terms and selection, can easily miss key areas of evidence. This is particularly the case with respect to social science and policy literature which may fail selection criteria despite meeting high standards in terms of rigour and quality in their fields. Quantitative or randomized control test (RCT)-based standards will overlook work that focuses on the contextual and situated processes that affect risks. Social science research’s specificity, in-depth understanding of practices and ability to ground those practices within their relevant cultural, economic and political situations are essential to any assessment of the origins and impacts of emerging infections [26] and the role of people and communities in mounting effective outbreak responses [27]. The view from molecular biology and other bioscience-related disciplines is of course crucial in any assessment of past and future infectious disease risks, but health, disease and recovery transcend any contagionist view of life [28–30]—they require us to understand the configuration of bodies, economies and social forms of life.

Bartlett *et al.* [9] acknowledge important gaps in their analysis (including the effects of climate change on production systems and zoonoses risk). Even so, it is the range of bracketed social and economic issues that merits careful attention. For example, what are the economic and business dynamics of livestock intensification and is it reasonable to assume that these will have little or no effect on production quantities, qualities and market structure? How can political economy and socio-spatial analysis be used to assess the impact of intensified systems on local and distant land uses and landscapes? What is the evidence that greater intensification of meat production reduces human–animal contact? It is to these questions and their implications for EID risk that we now turn, starting with an overview of industrialization before working through issues of political economy, framing livestock production, biosecurity, human labour, risk and regulation, and health vulnerabilities.

## 4. Industrialization and reindustrialization of livestock farming

Intensifying livestock production implies industrialization and reindustrialization of farm and food chain processes [31–44]. Industrialization refers to the adoption of production and management processes commonly associated with manufacturing, including processes of standardization, divisions of labour and applications of technology [40]. Reindustrialization in this context refers to the corporate-led reinvestment in and restructuring of livestock production practices following the first global financial crisis of this century [45,46]. The drivers of intensification and accompanying reindustrialization include increases in demand for cheap food, financialization, securing competitive advantage, new technologies and the opportunity to develop new markets [47,48]. Social science-led analyses of these drivers, their likely outcomes in terms of industrial expansion and growing livestock numbers and the extent to which these systems exert control over human, animal and microbial lives, or, conversely, serve to stretch those inputs and biological systems, is a key area for a system-wide EID-risk assessment.

Intensifying production requires capital investment and is made possible through private finance, integration with corporate firms and contracts, preferential loans from international development banks and state or trade block support in the form of grants and payments [49]. Servicing loans and investments often require drastic changes to produce (satisfying mass markets); orienting produce to growing markets with high demand; increasing specialization (focusing on one species or product line); and access to transport infrastructures, cold chains and processing. These changes have effects in terms of costs and profit margins per unit of production. They also tend to expose the farm business to a wider field of competition [50]. Once industrialized, this competitive environment fuels continuous improvement, output growth and productivity gains.

The need for investment and competitive advantage promotes the financialization and consolidation of livestock production. Increasingly, farms are integrated within large corporate entities, and a handful of transnational corporations (TNCs) now dominate global animal genetics, management systems, and national and international markets [51]. These corporations are vertically and horizontally integrated and organize all aspects of the industry: managing animal genetics; specifying and matching meat quality to international market conditions; developing, sourcing and supplying animal feed; stipulating medicines and vaccines; organizing grow-out conditions; orchestrating slaughtering and processing; and managing product lines and marketing [32,34]. The companies span international jurisdictions, often making use of locational differences in costs and regulations to grow livestock, manufacture products and supply markets at least cost. The result is a spatially complex economy wherein the stages of production (be that breeding, growing, slaughter and processing) tend to be organized within an integrated process that is segmented in terms of specialisms and locations. There are at least three implications (with EID-relevant consequences): (i) a tendency to expand output, (ii) a market-driven focus on reducing costs and factors of production, and (iii) a tendency to operate at both biological as well as economic margins.

First, although it may be heuristically reasonable to compare two systems of production in terms of yield per animal, it may be unrealistic to assume that changes are neutral with respect to output and markets. Any reduction of EID risk *per unit of production* will be offset by the need for significant expansion of, and extraction from, global livestock biomass intrinsic to the business dynamic of corporate integration and intensification. In other words, intensification is not simply demand-led; it involves the shaping of future demand for meat and other livestock products. In that sense, it tends to expand livestock numbers.

Second, the requirement to reduce costs has a number of consequences that can vary depending on location. In some parts of the world, and notably in North and South America, industrialization and reindustrialization have taken advantage of lifted restrictions on land-use change, waste processing and emissions. In the US pork sector, re/industrialization has seen a growth in mega-farms and further concentration of production in the mid-West and Carolinas, with notable environmental degradation [42] and human health concerns [52]. Feedlot growth in South America has benefitted from relaxed land-use restrictions and dispossession of indigenous groups (as well as growth in animal movements to markets in North America and Asia) [53]. An abundant supply of migrant labour has led to the concentration of production in areas of rural deprivation, where regional or state governments have relaxed labour laws in order to attract investment [42]. Labour supply has been a major comparative advantage for many producers in Asia, with post-COVID and post-African swine fever (ASF), industrialization taking advantage of reduced wage bargaining power in rural prefectures in China [38,54]. As firms take advantage of regulatory concessions and associated cost savings, there are likely to be consequences. As we detail in subsequent sections, land-use changes as well as labour conditions will alter the EID risk landscape.

Third, industrialized animals are increasingly bred for and required to service a standardized and optimized production process. Many farms specialize in specific elements of the product cycle (so breeding, multiplying, growing out, slaughter and processing) with increased livestock movements, flow of material produce, knowledge and capital within this integrated system relying on standard inputs (feed, medicines) and outputs (in terms of animal and product specifications). This standardization is a key element of the industry—it is important in terms of economies of scale for businesses that benefit from uniformity of product and production knowledge, and whose infrastructure (from breeding pyramids to processing plants) runs efficiently with minimal biological and or material deviance. In short, intensive animals differ from extensive animals in more ways than genetics. Form, growth rates, appearance, fat ratios, reproduction rates and so on can affect the demands on them and on the workers who raise, tend, slaughter and process them. As we detail later, the effects on animal health, vulnerabilities, conditions of production and human health may have EID consequences.

It is often assumed that industrialization is consistent with lowered EID risk (greater control of systems, higher biosecurity in terms of reduced incursion of pathogens and internal segmentation of livestock). And yet, the combined effects of sector expansion, environmental degradation (which can affect a wide area and has effects on microhabitats, ecotones and further afield), labour practices (which can produce new forms of human–livestock interactions), animal movements and high throughput animals (which can increase disease vulnerabilities) can have implications for EID risks. We expand on each of these in subsequent sections.

## 5. Political economy

Industrialization and reindustrialization, and associated investments, produce a range of intended and unintended outcomes that unsettle the zero-sum comparison of high-/low-yield production systems. Insights from political economy (studies of economic systems and their governance) and industrial geography (the study of spatial economies and industrial processes) [32] highlight the range of outcomes that result from agricultural intensification [34]. There are at least three issues to note here: (i) differentiation within the intensive or industrialized sector; (ii) coexistence and codependency of intensive and more extensive systems; and (iii) the displacement effects of intensification on the rural and peri-urban economy. We take each in turn and draw out the implications for EID risks.

First, industrialized farms tend to exist in complex networks of internally differentiated production. One manifestation of industrialization and improved productivity is the large, capital-intensive farm with improved closed-cycle systems (in pork production this is the farrow-to-finish farm). However, more common is the vertically and horizontally integrated system involving linked firms and multiple farms servicing components of the production line [32]. The resulting production landscape coheres to models within industrial geography [55]. The latter suggests a more heterogeneous and locally circumscribed pattern of farms and services than the intensive/closed, extensive/open dichotomous model. In some parts of the world, contractual intensification (where farms engage in intensive production as part of an overall process) is much easier to implement than closed-cycle farms. In the United Kingdom, for example, ‘Bed and Breakfast’ pig farms involve a corporate integrator supplying piglets, feed, technical advice and medicines to medium-sized farms. The farmer provides the buildings, land and labour, and contracts to deliver the pigs at slaughter weight by a specified date. The integrator benefits from standardized production, low fixed costs and the distribution of production risks across multiple grow-out farms. Broiler production is similar. With specified feed rations for each grow-out stage, and the requirement for centralized breeding, slaughter and processing facilities, spatial clustering of grow-out farms within a 40 km radius of the feed distributor and processor is the norm [33]. The result is a proliferation of middle-sized farms in a compact area. The key point here is that the *form* of industrialization is shaped by economic efficiency, risk management and expediency with respect to land-use politics and norms. The result is an internally differentiated landscape of industrialized holdings, serving facets of production (breeding, hatching, growing out, slaughtering and processing), with the transit of animals (and sometimes staff) between settings.

Second, intensive systems tend to depend on other intensive and more extensive systems. In the United Kingdom, for example, outdoor and indoor pig production systems established close cooperation links and shared innovation from the outset [56], and in recent years, it is common for outdoor-reared animals to be finished indoors (the finished pigs are often sourced from outdoor farms which have a comparative advantage in parts of the United Kingdom where grain production is less profitable) [57]. In the United States, intra- and inter-state cattle movements are associated with maximizing production in the beef and dairy sectors [58]. Some of this movement is related to climate- and drought-related drivers, moving cattle to available pasture and water. Some relates to the geographical separation of rearing and finishing operations [59]. There is also economic interdependency between intensive dairy and ‘dairy beef’ and veal producers, as surplus dairy calves are transported to feedlots [60]. The latter can involve long distances (over 1000 km) between the Upper Mid-West dairy operations and the Southwest where feedlots predominate. Finally, the fixed provision of slaughter and processing infrastructure necessitates large numbers of animal transfers [61]. As the 2024 widespread occurrence of avian influenza in US dairy cattle suggests, the long-distance movement of cattle between intensive dairy operations, and potentially back into beef production, can be epidemiologically significant [62].

Third, intensification reconfigures rather than replaces existing agricultural activity [63]. In a series of detailed studies across Southeast Asia, researchers found that the drive to commercialize poultry production initially favoured a raft of small- and medium-scale peri-urban operations that serviced the growing urban and middle-class appetite for chicken meat and eggs [64]. As those smallholders and peri-urban enterprises became economically squeezed by large agribusiness, informal networks and in some cases, illegal-trading practices proliferated [65]. A similar tale emerged in Egypt, where state- and corporate-led investment in large-scale indoor production of intensive poultry was economically dependent on both integrated production and processing, and non-integrated sales of broilers to small farms and eventually live bird markets. Rather than eliminating small and backyard farms, intensification was financially conditional upon established forms of production and consumption [66]. In China, there is evidence that the rationalization of pig and poultry production during the post-Mao second leap displaced small livestock farmers who turned to profitable farming of wild animals [67]. Industrialization of poultry pushed many farmers to breed wild geese, arguably fuelling the first major waves of highly pathogenic avian influenza [68]. In other settings, displacement of pastoralists generates new forms of ownership and risks. Indeed, extensive and pastoral systems have traditionally been low-risk systems, with relatively limited levels of human contact and closed herds (with few transfers of stock). These systems are changing in many parts of the world, with increasing concentrations of animals around small towns/water points, less transhumance and changing patterns of ownership as former pastoral owners sell up to absentee owners living in large cities who manage their herds from a distance with hired labour [69].

These analyses of internal differentiation, coexistence and reconfiguration suggest that the impacts of intensification are at best uncertain and at worst may contribute to EID risk. Intensification alters rather than replaces the mixture of production types and practices, and may well lead to market segmentation, increased animal movements, growth in irregular trading and greater complexity. As Bartlett *et al.* note, the resulting mixed landscape is considered the worst of all possible worlds in terms of EID risk [70,71]. Counter to their view, these landscapes are a product of, rather than erased by, investments in intensive production.

## 6. Framing livestock production

A founding myth of industrialized agriculture is the spatially self-contained, closed-system production unit, one that can regulate animal and microbial movement on and off farms. This framing of production neglects the footprint, overflows and inevitable leakages that characterize animal rearing (or indeed any economic activity [72]). Here, we raise matters of inputs, outputs and ecological disturbance before troubling the closure model of biosecurity in the next subsection. The role of social science, in this case, is to question analytical frames of reference and to ensure that the material spatialities of livestock production, and the inequalities they can reproduce, are carefully elicited and assessed.

All farms are essentially open systems, requiring water, feed and other inputs [73,74]. The footprint for extensive pasture systems may be roughly equivalent to the area of the farm (if emissions are ignored), but more intensive systems are likely to involve more intricate and extended resourcing. Grass-fed, zero-grazed beef production clearly requires associated pasture. Industrial aquaculture requires wild catches of feedstock and/or other sources of protein-rich feed [75,76], many of which can contribute to environmental and nutritional consequences in extraction and export regions [77]. China’s pig industry has an enormous appetite, importing 28.35 million metric tons of soybean meal from the United States, Argentina, Brazil and Ukraine, with associated environmental impacts in terms of habitat loss and land degradation [78]. The contribution of livestock industries to the clear-cutting of forested landscapes and the effects on emerging and re-emerging vector-borne diseases have been modelled by economic geographers [79].

Other inputs include medicines, and, notably the large-scale use of antimicrobials in treating and preventing infections as well as promoting livestock growth [80]. Intensive production, and the resulting need for enhanced infection control in densely packed, fast-growing populations, often result in high levels of antimicrobial use (and even where this use is reduced per unit of production, the total uses and impacts can be sizeable). The production of active pharmaceutical ingredients (largely in Asia), the manufacturing-related pollution, and the large quantities used in farming contribute to the selection, persistence and transmission of antimicrobial-resistant bacteria, with consequences for the future sustainability of human as well as animal health treatments [81]. Human health systems rely on effective medicines, and the use of over half the world’s production of antimicrobials in livestock systems, much of it in industrial settings [80], is a major cause for concern. Even in states where antimicrobial uses in livestock are increasingly regulated, the volume of treatment uses, their potential impact in terms of environmental resistance, and, in some cases, their partial displacement by resistance-conferring disinfectants, are testimony to the difficulties of managing the problem [82].

There are other inputs that are rarely considered but can contribute to EID risks. In the hormonal management of porcine reproduction, for example, successful insemination on farms is reliant on pregnant mare serum gonadotrophin (PMSG), which is extracted on South American horse blood farms [42]. The latter operate outside regulatory and veterinary oversight on semi-wild privately owned forests where horses are impregnated, have blood extracted on a weekly basis in the last months of pregnancy and are then assisted in aborting the foals [83]. The impact of the intensive operation on landscapes thousands of miles away, and on potential emerging infections on those farms, is an important consideration of any model of risk.

Output-related challenges include the large volumes of litter and animal waste products produced in concentrated animal feeding operations. These may be stored close to the site and spread on the surrounding landscape in ways that can generate ecological degradation [10,84], human health hazards, and contribute to environmental and racial injustice [85]. In England and Wales, in the last decade, the proliferation of broiler farms in the Wye Valley to meet the demand of Cargill’s expanded processing capacity in the region has contributed to phosphate pollution of local soils and rivers through the spreading of litter from poultry houses [86]. The impact in terms of direct transmission of microbes to the environment is uncertain (though may be a significant component of the transfer of pathogens, antimicrobial-resistant bacteria and genes to the environment). However, the indirect effects may also alter disease systems. The ecological degradation of the catchment reduces available wild bird habitat, affecting feeding opportunities. Poor feeding leads to greater disease susceptibility, more mixing of wild bird populations, increased transmission possibilities, and a tendency for the wild birds to forage in or close to farm environments [87]. This disturbance and displacement of wild animals as a result of wider ecological effects of intensive farms can impact EID risks, including in this case a potential shift in the pressure on farms of high levels of highly pathogenic avian influenza (HPAI) in wild birds [67].

## 7. Biosecurity

Agricultural biosecurity is often defined as the exclusion and/or containment of harmful organisms [8]. The exclusion or closure model [88] can neglect several issues including changes to the environmental or infective pressure on farms; the capacity of farms to achieve closure; the effects on workers’ lives and rural landscapes and the changing vulnerabilities of systems where breakdowns are less frequent but magnitudes or costs are amplified [89]. We take each in turn.

First, any biosecurity assessment needs to include the spatial impacts of the farm. As we have noted, if farms contribute to environmental and habitat degradation, then this may have consequences for overall EID risk and heightened bio-insecurity (from ecological destruction of distant landscapes to impacts on farm microhabitats and ecotones). As infective pressure grows and as attack rates change through selection pressures (the H7N6 HPAI in South Africa in 2023 decimated the national flock and overran biosecurity measures), then a system of security that is based on imagined spatial closure becomes increasingly questionable [88]. Indeed, it may result in a ‘treadmill of purity’ [90], wherein farms are continually tasked with delivering unachievable levels of hygiene and containment that are beyond their means or control.

Second, as many empirical and operational studies have confirmed [88,91–95], agricultural biosecurity is a continuous (temporally extended) process that may reduce but does not eradicate risk. No system is failsafe and there are many cases where indoor production facilities with ostensibly good biosecurity suffer routine breaches (they include pinch points in production including thinning and harvesting in broilers; damage to buildings following storms; food and bedding delivery and storage; staff shortages and sharing of labour; ventilation systems; vermin control and so on). Many farm businesses find the costs of maintaining continuous vigilance to be debilitating. European farm buildings can be old and costly to maintain. In the United States, large farms tend to be open-air concrete structures with netting to obviate the need for air conditioning. In subtropical environments, farm biosecurity is always balanced against the need to reduce overheating of animals. The result is a far from bio-contained environment.

Third, restrictions placed on farms and farm workers can have a number of negative consequences [96]. Biosecure farms that eradicate soft surfaces, trees and water features from their surroundings may reduce wildlife presence but can become more vulnerable to climate change risks (floods and extreme temperatures, both of which can increase EID risks). If farms remove wildlife-friendly landscape features they can violate local planning regulations but also reduce income opportunities associated with tourism and leisure. Workers on biosecure premises can become increasingly restricted in terms of on-farm roles and off-farm lives. In the United States, pork-sector workers were discouraged from residing and socializing with acquaintances from other parts of the industry in order to prevent disease transmission [42]. In China, labourers in large pig operations can be expected to live for long periods (up to two months) in a securitized farm-based compound, as working conditions become increasingly carceral [97,98].

Finally, while biosecurity measures tend to focus on reducing the frequency of pathogen incursions, less attention is afforded to the consequences of incursion (or magnitude). If capital outlay on biosecurity is offset by expanding output and throughput, animals may become more susceptible to disease [7,88]. In these circumstances, what looked like a highly contained process turns out to harbour potentially explosive leaks [99]. The large social science literature on normal accidents (where there are many complex pathways, and the tendency for small failures to cascade and lead to catastrophic breakdowns) is apposite here [100–102]. Other work that utilizes actor networks to emphasize the generative nature of complex social, material and political relations is also an important source of social science insight [64,88,89]. And, as industrial systems grow, they may be subject to new kinds of security risk. They include new disease risks generated within industrial systems (e.g. protein cycling that contributed to bovine spongiform encephalopathy) as well as the enhanced security risks of large, contained animal populations. For example, in China, there are reports of drone-borne ASF attacks on large pig units. The perpetrators are thought to benefit from sudden shifts in pork commodity prices subsequent to ASF affecting large herds [103].

## 8. Human labour and human–non-human contact

Intensification is frequently assumed to involve a progressive reduction of on-site human labour through the adoption of mechanized processes (automated feeders, remote monitoring, animal sensors, robotic extraction and artificial intelligence all reducing the need for close animal contact) [104,105]. The implication is that there will be fewer human–animal contact events and lower EID transmission risks. And yet, this assumption can be overstated. As already noted, industrialization and reindustrialization have depended in some parts of the world on the availability of low-cost labour (in lower- and middle-income countries (LMICs) but also in areas of rural deprivation with exploitable migrant populations in higher-income countries including the United States and elsewhere). Many are employed in slaughter, cutting and processing plant which are labour intensive (and highly gendered as well as racially structured [106]). The implications of treating animal processing operations as vital infrastructure during the COVID-19 pandemic became a major public health issue [107]. Even prior to slaughter, industrial farming more generally involves new forms of human work. This may relate to the economic and scalar interactions of expanded farm size and production pinch points. In poultry meat production, it involves the labour necessary to thin and harvest broiler houses—a pinch point that requires rapid and careful handling by skilled depopulation teams to meet stocking regulations and to deliver just-in-time birds to the processing plant [7,108]. While these practices are being displaced in some countries by machines, in those parts of the world with low labour costs, human contact with stressed avian bodies in densely populated houses and time-limited conditions are more rather than less common.

Other forms of labour are necessitated by high-yielding animals exhibiting higher mortality rates, more metabolic disease and infertility problems. In general, the more those livestock animals are pushed to their productive limits, the greater the need for human labour to compensate for their reduced function. Pig rearing, for example, is increasingly characterized by new forms of human labour that compensate for the over-calibration of porcine life. Hyper-prolific sows, favoured by the large corporate livestock systems, produce litters that exceed their functional teats. Piglets receive less immunity-conferring maternal colostrum and industrial runting rates increase. In breeding facilities, there is an expansion of human labour in the form of care work to maximize piglet survival and make sure weaned pigs maintain growth rates [42]. Even in grow-out farms, farmers can be expected to care for weaned pigs for longer, with farms specifically selected for the placement of animals where corporate managers know that the farmer has a record in providing quality animal care (close attention and nurturing of stock). Far from removing labour, corporate agribusiness and intensive livestock production continue to take advantage of self-exploitative forms of work [109].

Other pinch points include vaccination and loading animals for transit when the ideals of low human contact cannot be safely met. In intensive pig production, the industrial norm of artificial insemination can also generate new forms of human–non-human animal contact and potential disease transmission. On these farms, it is common for sows to be ‘mounted’ by farm workers for up to an hour as workers encourage the sow to receive the semen [110]. Even where companies have moved to intra-uterine insemination using backweights, there is contact in terms of inserting catheters and making observations until the process is judged to be complete. Close human contact with animals is a condition of reproduction in a modern pig breeding unit [111].

From an EID risk perspective, the dynamics of interspecies contacts are clearly different between extensive and intensive systems. While quantities of human–non-human animal interactions may be significantly reduced in shifting from 100 extensive farms to a single large producer (though see earlier section on political economy and displacement), intensive systems do not obviate labour. The squeezing of margins involves reconfigurations of the human–animal interface and may generate new and possibly dangerous forms of contact and even EID ‘take-off’. Assuming intensive production involves low or zero labour is incorrect. A key task for social science contributions to EID risk analyses is to investigate labour practices and potential for transmission within intensive livestock systems in various jurisdictions.

## 9. Risk and regulation

As systems commercialize and intensify, and as risks grow (with greater numbers of animals in close proximity), it is conventionally assumed that pathogen control and disease risk management will compensate accordingly and even improve. Risk management at integrated, high-throughput farms is often codified by integrators, processors and upstream retailers who provide guidance on everything from worker conduct to medicine use, specification of buildings and standard operating procedures [112]. Much of this is audited and written into conditions of production and contractual obligation [82] and subject to industry- as well as state-based regulation [108]. Similarly, there is the common claim that many of the unsolved risks that pertain to intensive systems can be overcome with the application of technology [113]. Genetic editing, improved digitization and datafication of farm environments, better herd management and so on are often cited as solutions to concerns around genetic vulnerability, disease threats, farm animal health and welfare [113]. There is an assumption, too, that many of the issues with EIDs can be solved with resource transfers from high- to low- and middle-income countries [114]. However, this regulatory codification and optimism regarding technology and its transfer can be tempered by a number of counter-tendencies that require social science investigation and input.

The extent to which risk management practices are fulfilled will depend on the social and economic context of the livestock setting and the disposition to risks. For example, farmers and farm workers can become fatalistic, complacent or even disinterested in disease risks. If breakdowns occur in ways that have little relation to effort expended, if farmers feel that disease drivers are beyond their control, or if the on-farm costs of disease are relatively minor, then farms may fail to install expensive controls or do so with reduced vigilance. In cases where zoonotic diseases of human health concern, or of economic or trading concern, have relatively little impact on livestock health or production, there can be a conflict between public and private goods. Where the stakes are high, the clash of interests can lead to delays in disease surveillance and control (examples include the 2009 swine flu pandemic that had little impact on pork operations, as well as the 2024 avian influenza outbreak in dairy in the United States, which had relatively minor effects on farm production but raised public and wildlife health concerns).

These dispositional risks will be influenced by the availability or otherwise of compensation. If compensation thresholds are too high, there may be reduced incentives to minimize risk, while low or zero compensation can reduce disease reporting, impede surveillance, and negatively affect the investment environment [115,116]. In LMICs, these risks and rewards may be particularly stark. For example, pig farmers in Myanmar mediated their reading of disease risk through their economic and environmental situations, their lack of available options and poor rewards for investment [117]. Aquaculture farmers in Bangladesh were similarly reluctant to give up on tried and tested forms of multi-crop and multi-species farming that insured them against export crop failure. In a subtropical climate, with variable monsoon and fluctuating markets, moving to intensive production increased rather than reduced livelihood risks [118]. The implication that there is a single model of farming and that this dictates a one-size-fits-all approach to food production was dangerous and neocolonial [119]. Optimization of livestock production was part of the problem and not the solution [114,120].

Organizational risks include the difficulties of managing risk within the system of production. For example, in Europe, rural labour shortages and staff retention problems can result in overstretched and time-pressured workers servicing multiple sites [52], with new and untrained workers skipping protocol. An under-researched issue is the effect of changing contract and industrial relations on risk practices. As farmers become the equivalent of labourers on their own land [34], there may be implications in terms of their ability to adapt to or indeed flag up new health challenges. In a parallel development, many veterinarians are increasingly directly employed by livestock companies and are closely aligned with production managers with healthcare attention shifting from animal bodies to herd/flock parameters [121]. Many are increasingly making use of tele-care and off-site advisory roles. The organizational balance between intensified codification of production and the ability of stock people and animal healthcare practitioners to manage their farms and animals appropriately remains to be investigated. Certainly, intensive, standardized and large-scale production may increase the ease with which herd or animal anomalies can be identified and managed at the herd level but also reduce the opportunities for observation of animals on the farm. The effects on responsiveness, emergency capacity and the standing reserve of skills necessary for nuanced diagnosis and early recognition of disease signs need to be investigated.

As social scientists have long argued, risk is a particular form of knowledge practice that emphasizes measurable unknowns [122] and tends to suppress other forms of incertitude (uncertainty, ambiguity and ignorance) [123,124]. There is a tendency in conventional, bioscience-led analyses to focus on the relatively measurable risk of pathogen transmission and neglect the uncertainties that surround production systems more generally. Indeed, one could argue that as a production system becomes more oriented to measurable external risk (disease incursion and disease freedom), then a failure to analyse system resilience, internal proliferation and general system failure becomes more pronounced [101].

Beyond the farm or livestock premise, there is the assumption that intensive farms are more likely to be subject to and adhere to state-based regulations. This assumption of regulatory control is undermined by understandings of regulatory regimes, regulatory capture, the locational practices of transnational corporations and the changing nature of global food systems. Indeed, corporate and commercial interests can influence as well as bypass state-based regulation or public health accountabilities. The difficulties in achieving transparency over transmission events in the 2011 swine flu pandemic, where workers in the meat industry may have been key in EID take-off, were testament to some of the governance issues at stake [125]. Market- and shareholder-based governance of large commercial companies are similarly unconvincing. Recent work on corporate financialization through institutional equity investors (universal owners) which in theory should accentuate environmental, social and governance (ESG) issues in the livestock and food sector, has tended to conclude that investor-led governance is at best arm’s-length and at worst tends to focus on reputational rather than operational risks [46,126].

Close ties between large food corporate entities and national elites also suggest regulatory capture, complicity in sanctioning new plant and the difficulty of decoupling interests [127]. We have already mentioned the intense lobbying of TNCs in seeking planning, labour, environmental and production concessions. However, there are also direct issues relating to EIDs. For example, trading states are keen to maintain disease-free status and may under-report notifiable as well as new diseases. COVID-19 was an interesting test case in the regulation and reporting of newly emergent diseases, but there are examples of livestock diseases being renamed or denied as a means to protect state and private interests [128]. To safeguard trading status, state departments may opt to limit vaccine use if associated testing is insufficiently robust to separate vaccinated and infected animals. Finally, as trade patterns shift from a bi-polar (North–South) pattern to a multi-polar model (with China altering the livestock production geography and flow of goods), and as domestic markets for meat grow in lower- and middle-income countries, the assumption of greater regulatory control in the world’s livestock systems may be misplaced [129].

## 10. Health vulnerabilities

Pandemic risk relates to more than the emergence and transmission of pathogens. The health status of potential recipients (hosts) is a necessary component of any epidemiological risk analysis. For COVID-19, outcomes in higher-income states were shaped by socio-economic inequalities, nutritional disparities and associated high levels of obesity, diabetes and cardiovascular disease [130]. As social science studies of Ebola have demonstrated, in landscapes where people have long cohabited with wildlife hosts, diseases do not simply spill over, they flare up, triggered by stresses and a composite of many factors that are quite different to those assumed by the contagion model [13].

In terms of livestock-related EID risk, there is a key issue here in terms of associated health changes once livestock production is industrialized. Intensive production systems tend to be troubled by the production of surplus and the resulting variations in farm-gate prices. A major means of managing surplus is to ensure sufficient markets for livestock products (a process called ‘venting’) [34,57]. The result is a proliferation of standardized products, many of which are incorporated into highly processed foods (consumed by people, other livestock and companion animals). While this reduces industrial waste, the economically necessary hyper-processing of at-scale production can have dietary and public health impacts [21,131]. While greater specification of fat quantities in meat can serve high-end markets, the economics of production require that most bodily substances from pigs, cattle, salmon and poultry are marketable. The effect on human diets in terms of the proliferation of newly processed foodstuffs may contribute to new forms of dietary malnutrition (or the triple burden of malnutrition in terms of poor food availability, over-nutrition and the consumption of micro-nutritionally poor-quality food [132]). The manufacture and saturation of foods with antioxidants and endocrine disruptors alters global (industrial) metabolisms [133] and may be contributing to levels of obesity and other metabolic diseases including diabetes. While product proliferation increases the diversity of output from livestock industries, it can also reduce dietary range through standardization, processing and the displacement of alternatives through manipulation of price points and consumer taste. We mentioned nutritional qualities in an earlier section in relation to pasture-reared beef, but the principles may be more general [134] and have consequences for public health. For example, shifts to commercial inland aquaculture in Asia reduced production and dietary intake of less marketable small fish, which were important in terms of essential oils and micro-nutrients [135]. Wild catches of marine-derived industrial animal feedstock off the coast of West Africa have similarly generated a dearth in the availability of nutritional food in Senegal and other coastal states [77].

In terms of EID risk, there is a need for social science knowledge to assess the implications of intensive farming on human and animal health. Who gets ill and why [136] is a clear and pressing issue for pandemic preparedness and one that relates to drivers of vulnerability that cannot altogether be decoupled from livestock farming [137]. If livestock becomes ‘saturated by economic value’ [42], then other bodies (including human ones) become biologically marked by that system of values.

## 11. Conclusions

Our survey of social science engagements with industrialization processes, political economy, spatial framing of farms, biosecurity, labour practices, risk regulation and health outcomes have material effects on the following risk factors. See table 2 which summarizes these insights (we have omitted rows where changes are negligible).

**Table 2.** Adjusted EID risks associated with intensification following social science integration. Red indicates increased risks, amber indicates greater uncertainty.

**characteristic**	high yield	low yield	effect on EID risk once higher-yield systems predominate positive (red), or more risk +, amber suggests great uncertainty.	comments (implied effects of intensification on EID risk)
biosecurity	low	medium	+	overall security can be compromised by greater vulnerability and network relations
livestock movements	high	low	+	intensification increases the volume of movements between specialized operations
population size	high	medium	+	gross product will expand in industrial and capital intense systems
density	high	low	+ (?)	density will be affected by industrial form and inter-farm relations
health and welfare	uneven	uneven	+ (?)	regulations, care and compliance are uneven and require social science investigation
habitat quality	low	medium	+	footprints and extended frames of reference indicate an overall reduction in habitat quality
ecotone extent	high	high	+	affected by livestock emissions and wastes
livestock contact with wildlife	low	high	?	degradation of ecotone can increase opportunistic and stressed wildlife on farms
human contact with livestock	medium	medium	?	the form of contact in highly stressed systems requires detailed study

In all cases, there is a shift in polarity and/or key questions surrounding the extent to which changes are consistent with the hypothesis that intensive production will reduce EID risk. Here, we briefly summarize our assessments with emphasis on the role of social science in future integrated assessments:

**Biosecurity**—If biosecurity is redefined to include both biocontainment *and* the vulnerabilities associated with production at scale and close to biological margins; the propensity for normal accidents and systemic failures; the ecological disturbances that can change emergence, persistence and transmission dynamics; then intensive livestock production results in greater EID risk. We would suggest that biosecurity becomes an empirical matter where the question is not so much good or bad biosecurity (based on the presence of barrier systems and standard operating procedures) but an overall assessment of system-wide security. This would involve a composite assessment that is applied beyond individual farms. There are clear roles here for social scientists to study the economic, behavioural and broader system challenges involved in securing livestock systems, and to consider the implications of, and barriers to, changing spatial frames for security in terms of legal, trade and other systems.

**Livestock movements**—the assumption that livestock movements are reduced in high-yield systems is challenged by the heterogeneity of industrial systems and political economic analyses. A key role for social scientists in any risk assessment is to apply socio-economic analyses and insights to empirically verify and offer foresight in terms of how intensification impacts the rural landscape and economy. This will be variable and will depend on in-country expertise to assess the likely form of industrialization and its political and economic repercussions.

**Population size**—while the heuristic value of comparing intensive and extensive systems in terms of fixed total output is useful, social scientists are required to assess the impact of intensification on farm businesses and markets. Intensification will tend to require changes to market preferences and an expansion of demand (including for meat and livestock-related product lines). Analyses of the market dynamics of intensification, and how livestock companies shape market and product demand, are required. Only then can livestock numbers and the health impacts of expanding consumption be estimated.

**Density**—social scientists need to be involved in understanding the forms of intensification which can produce varying outcomes in terms of animal and farm density. Specialization and locational efficiencies can generate new landscapes of production where EID risks are increased.

**Health and welfare**—there is wide variance in terms of the health status of animals across all sectors and within as well as between intensive and more extensive systems. Social science is required to investigate the pressures on animal health in various systems. The role of and compliance with regulatory processes, the structure of commercial operations and their effects on contract or other operations, are topics which require social science insight and analysis. The effectiveness or otherwise of shareholder-based governance on welfare standards and the tendency to rely on quantified measures of welfare and health need to be assessed. The changes in animal sensing and veterinary observations associated with large-scale animal production require social science study and insight.

**Habitat quality/ecotone extent**—livestock systems are open systems, with impacts at local and non-local sites. Social and economic scientists should be involved in global assessments of livestock systems, understanding the negative consequences of resource flows and highlighting externalities. The social and environmental justice components of livestock production require social sciences to engage with impacted communities and to ensure that environmental and health degradation are not shrouded by racialized, epistemic or other forms of systematic injustice.

**Human–animal contacts**—intensification changes rather than reduces human labour requirements and practices. Livestock systems can have varying amounts and forms of human–livestock contact, making it an empirical matter for social science investigation. The stresses placed on human and non-human bodies associated with industrial processes at all stages in the product cycle can be elicited through detailed empirical work including ethnographies of workplaces and production sites.

Finally, while not part of the initial table, regulation is a key area for social science investigation. The extent to which corporate owners and state-based sponsors control production and processes requires detailed study. This is an empirical matter and requires insight into social and economic organization and analysis of the exigencies and limitations of social and economic power. The requirement for high throughput systems to push economic and biological margins to their limits and the tendency for systems to operate close to critical thresholds should be key topics for social science input.

Disease is always more than a matter of pathogen transmission, contact and contagion—it requires us to combine the best knowledge we have of microbial processes, virulence, host dynamics and ecological processes with knowledge of cultural, social and economic lives, their role in shaping businesses, human practices as well as reconfiguring animal lives. These are proper to what elsewhere have been called disease situations [7], the meeting up of microbes, political economies, knowledge, cultures and various other entities and that need to be thoroughly analysed along with scientific assessments if we are to judge future risks and establish reasonable and realizable pathways to their mitigation. Models are essential tools in terms of generating questions for future research and policy [138], but they must be developed in ways that are cognizant of social science explanation and understanding.

## Data Availability

This article has no additional data.
